# Atropine reduces aldicarb-induced sensitivity to *C. elegans* electroshock model

**DOI:** 10.17912/micropub.biology.000621

**Published:** 2022-08-08

**Authors:** Nirthieca Suthakaran, Trisha Brock, Akshay Naraine, Paola Gonzalez- Lerma, Chris Hopkins, Ken Dawson-Scully

**Affiliations:** 1 Department of Biological Sciences, Florida Atlantic University, Boca Raton, Florida, USA; 2 InVivo Biosystems, Eugene, Oregon, USA; 3 Department of Psychology and Neuroscience, College of Psychology, Nova Southeastern University, Fort Lauderdale, FL ;; 4 Department of Biological Sciences, Florida Atlantic University, Boca Raton, FL

## Abstract

Atropine has been used as an established anticonvulsant treatment for nerve agent intoxication. Atropine reduces electroshock recovery time among aldicarb-exposed wild-type *C. elegans* .

**Figure 1. Atropine reduces electroshock recovery time among aldicarb-exposed animals.**
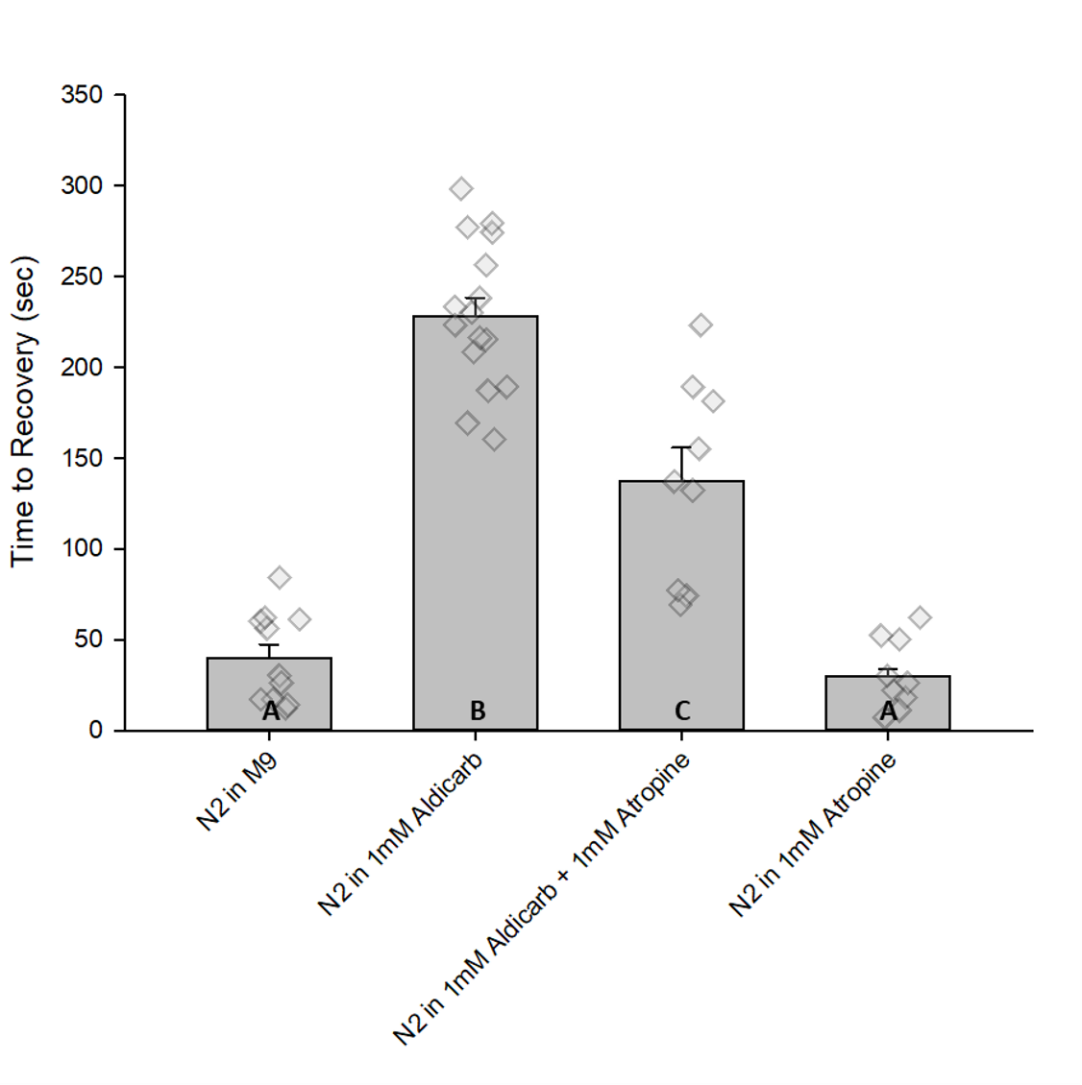
Average times to recovery are shown for N2, wild-type, *C. elegans* with overlaid individual data points for electroshock assay (total M9 n = 11; aldicarb n = 16; atropine n = 18; aldicarb + atropine n = 9). Wild-type worms were treated with M9 saline, 1mM aldicarb, 1mM aldicarb + 1mM atropine, and 1mM atropine. M9 was used a sham condition. 1mM aldicarb was used a negative control, and 1mM atropine was used as a positive control. Recovery time was defined as the point at which three normal sinusoidal wave-like swimming movement resumed following the initiation of the shock. *C. elegans* exposed to M9 sham condition averaged 39.9 seconds to recover. Worms in 1mM aldicarb averaged 228.25 seconds to recover, and worms in 1mM atropine averaged 29.74 seconds to recover. Worms treated with both atropine and aldicarb exhibited recovery in 137.44 seconds. (Aldicarb + atropine = 137.44 ± 18.5 sec; aldicarb = 228.25 ± 10.2 sec; mean ± SEM; p < 0.05). Data are means ± the standard error of the mean (SEM). Statistical significance (p < 0.05) was designated using letters where different letters show statistical differences, and the same letter indicates non-significance; one-way ANOVA and Student-Newman-Keuls posthoc analysis. Videos of the assay were recorded for 5-minute durations.

## Description

Electroshock has been used to study seizure in invertebrate and vertebrate models. Whereas the maximal electroshock seizure test, or MEST, has been considered the gold standard for testing seizure in rodent models, our lab developed an electroshock assay to investigate seizure-like behavior in *C. elegans* (Tolman et al.,1946; Rogawski et al., 2003; Risley et al., 2016; Suthakaran et al., 2021). We define the seizure behavior characteristics in our assay as paralysis, hypercontraction of muscles, and unilateral body bends which is similar to the description of seizures in other models (Tolman et al., 1946; Williams et al., 2004; Marley & Baines, 2011; Risley et al., 2016). FDA-approved antiepileptic drugs have shown efficacy in our assay, making it a rapid screening tool for potential novel treatments (Risley et al., 2016; Suthakaran et al., 2021).

Aldicarb is categorized as an agent causing irreversible AChE inhibition and may be fatal, depending on the level of exposure, if not treated immediately (Cavalcante et al., 2020). Aldicarb application causes hyperexcitability, excess muscle contraction and eventual paralysis in *C. elegans* as a result of acetylcholine accumulation at the neuromuscular junction (Opperman&Chang, 1991; Mahoney et al., 2006; Spensley et al., 2018; Giles et al., 2019). We previously examined aldicarb-sensitive mutants *eel-1* and *ogt-1* in our electroshock assay and observed delayed recovery (Opperman et al., 2017; Suthakaran et al., 2021). This observation, and the known effects of aldicarb at the neuromuscular junction, led us to predict that aldicarb application should delay recovery from electroshock, and this is indeed what we observe (Fig. 1).

Atropine exerts its effect postsynaptically by blocking muscarinic ACh receptor (mAChRs) activity (Eddleston et al., 2002; Eddleston et al., 2009; Arnot et al.,2011; Michael et al., 2015; Lochner et al., 2016). Atropine is most commonly used in cholinergic pesticide poisoning but is an established anticonvulsant treatment for nerve agent intoxication (Shih & McDonough, 2000; Eddleston et al., 2008). Previous findings have shown that atropine delays recovery from aldicarb-induced paralysis (Spensley et al., 2018). We therefore expected that atropine might delay recovery from electroshock and/or exaggerate the prolonged recovery time observed with aldicarb exposure. However, atropine alone did not impact electroshock recovery, and surprisingly, atropine partially suppressed the effect of aldicarb on electroshock recovery time (Fig. 1).

Our hypothesized biological mechanisms for atropine’s antidote effect is that when aldicarb inhibits acetylcholinesterase and results in excess acetylcholine, atropine then binds to post-synaptic mAChRs and reduces the acetylcholine-induced activation. Recent findings suggest that atropine does not rescue effects of aldicarb, but there are two key differences between these studies and ours (Spensley et al., 2018; McCulloch & Jin, 2020). These other studies used different convulsion assays that analyzed different types of baseline movement and also used greater concentrations of aldicarb, which would result in faster and more widespread inhibition of acetylcholinesterase enzymes. Our electroshock assay may be more sensitive as a lower dosage of aldicarb can be used since the primary stressor is the electroshock and not aldicarb itself. Since we used 4x less aldicarb in our assay and the same amount of atropine (Spensley et al., 2018), we may have uncovered a potential antidote effect for atropine.

## Methods

*C. elegans* electroconvulsive seizure assay: For the experiments in this study, wild-type animals were habituated in M9, 1mM Aldicarb, 1mM Aldicarb + 1mM Atropine, and 1mM Atropine solutions for 30 minutes prior to electric shock. All *Caenorhabditis elegans* strains were run in the electroshock assay (Risley et al., 2017). The experimental set-up included Grass SD9 stimulator, Grass SD44 stimulator (utilized as a 3 second timer), dissecting microscope with a camera (Hitachi model KP-D20BU), twelve-inch television monitor, and an HDD and DVR recorder (Magnavox model MDR535H/F7). In 10 mm long clear plastic tubing (Tygon® microbore tubing), 15μl of M9 saline solution was added. Roughly three to six 1-day-old adult worms were picked using a platinum wire pick and placed in those plastic tubes, after which they were incubated for 30 minutes. After incubation, an insulated copper electrode was inserted to both ends of the plastic tube. A 1cm gap was measured in between both electrodes. The electrodes were fastened with alligator clips to a square-pulse generating stimulator (Grass, SD9) that delivered a 3 second, 47V shock.

Electroconvulsive seizure assay video recovery time recording: Video capture via a dissecting microscope camera (Hitachi model KP-D20BU) was initiated 10 seconds prior to the administration of the shock. Synchronized adults (L4) had a baseline behavior recorded for 10 seconds prior to administration of the electric shock. Speed was normalized to M9 buffer to examine the effects of a given genotype on locomotion. The mean speed was calculated every minute and corresponded to the total amount of sinusoidal wave-like swimming per worm. Video capture continued for up to 5 minutes after the shock had taken place. Due to electrolysis, bubbles formed on either end of the plastic tube. Data points corresponding to the recovery times of each *Caenorhabditis elegans* were then collected. Recovery time was defined as the point at which three normal sinusoidal wave-like swimming movement resumed following the initiation of the shock. Each individual worm was manually scored the minute they resumed sinusoidal motion. *C. elegans* were excluded from analysis if they intersected the peripheral bubbles. All recovery times upon electroshock are shown as averages for each genotype.

Pharmacological manipulations: Drugs were dissolved directly into M9 saline solution and 15μl of solution was aliquoted into transparent plastic tubing. Animals were incubated in drugs of interest for 30 minutes prior to electric shock. The drugs tested were Aldicarb (CAS: 116-06-3) and Atropine (CAS: 51-55-8).

## Reagents

Strains: N2

Chemicals: Aldicarb (CAS: 116-06-3), Atropine (CAS: 51-55-8)
